# Editorial: Eco-smart biocontrol: harnessing microbe-plant synergy for sustainable agriculture

**DOI:** 10.3389/fmicb.2026.1965755

**Published:** 2026-09-11

**Authors:** Dharmendra Kumar, Durgesh Kumar Jaiswal, Jay Prakash Verma

**Affiliations:** 1 ICAR-Central Potato Research Institute, Shimla, India; 2 Graphic Era University, Dehradun, India; 3 Banaras Hindu University, Varanasi, India

**Keywords:** biocontrol, climate change, consortium, microbiome, sustainable agriculture

Sustainable agriculture is increasingly shifting from reliance on chemical inputs to biologically based approaches that harness the complex microbial systems in plants and soils. Overuse of chemical pesticides can harm the environment and drive resistance in plant pathogens, while intensive cropping can disrupt soil biological functions and weaken ecosystem resilience. In this context, beneficial microorganisms are a vital resource for developing alternative methods for disease control, enhancing plant growth, managing nutrients, and improving soil health. Despite their promise, applying microbial biocontrol in real-world agriculture remains difficult because microbial effectiveness depends on plant genotype, soil properties, microbial community structure, environmental factors, and farming practices. The articles in this Research Topic, *Eco-smart biocontrol: harnessing microbe-plant synergy for sustainable agriculture*, offer diverse insights into these issues, from manipulating soil microbes at the field level to analyzing the genomes and transcriptomes of individual biocontrol agents.

The central concept of eco-smart biocontrol is that plants function as integrated holobiont systems that interact closely with complex, dynamic microbial communities. Beneficial microorganisms can promote nutrient acquisition, plant growth, stress tolerance, immune responses, and disease suppression, while plants, in turn, actively shape the composition and assembly of associated microbial communities in their surrounding environment (Pizzi et al.; Reddy et al.). Therefore, successful biological control is increasingly viewed as an ecological process rather than the application of a single microorganism.

Contributions to this Research Topic showcase this principle across diverse agricultural contexts. Chiuta et al. demonstrate how short-term crop rotations, such as sorghum-potato and *Cucumis africanus*-potato, improve soil organic carbon, microbial diversity, and enzyme activity compared with monoculture, highlighting crop rotation as a microbiome management strategy that supports soil health and pest control. The sorghum-potato rotation also progressively improved soil structure indicators. These results show that crop

rotation serves not only as an agronomic technique but also as a way to shape the biological environment in which beneficial and antagonistic microbes thrive. Consequently, this expands the idea of biocontrol from merely applying microbial products to adopting management practices that foster a healthy, diverse soil microbiome.

Anveshitha et al. explore microbial consortia that degrade lignocellulosic rice straw into a nutrient-rich soil amendment, linking biological resource recovery with soil fertility and climate change mitigation.

Gaur et al. discuss moving from single microbial inoculants to synthetic microbial communities (SynComs) for biocontrol, which can better account for colonization, interactions, and environmental factors influencing disease suppression, aligning with efforts to engineer microbiomes rationally. The authors highlight that plant health and disease control result from complex interactions between plants and their microbial communities. While microorganisms like *Bacillus, Pseudomonas*, and *Trichoderma* show significant biocontrol potential, their effectiveness can vary in real-world settings. Synthetic microbial communities offer a strategy to combine organisms with complementary functions, such as producing metabolites, forming biofilms, colonizing plants, and modulating immune responses. This consortium-based approach is especially important for developing more reliable biocontrol methods, as it emphasizes that microbial cooperation and functional complementarity are as crucial as the activity of any single organism.

Di Rico et al. provide mechanistic insights through genomic and transcriptomic analyses showing that effective biocontrol involves secondary metabolite production, transcriptional reprogramming, and stress response, emphasizing the importance of dynamic microbial responses for successful antagonism. The article offers key mechanistic insights into how *Pseudomonas* sp. PF05 acts as a biocontrol agent against *Fusarium oxysporum*. Comparative genomic analysis revealed that the highly antagonistic PF05 strain has an expanded range of genes for producing secondary metabolites, including phenazine, polyketide, and non-ribosomal peptides. Nevertheless, transcriptomic data indicated that its biocontrol activity is not solely driven by traditional antifungal pathways. During interaction with *F. oxysporum*, PF05 undergoes coordinated metabolic and regulatory shifts involving efflux, detoxification, stress response, and metabolic reprogramming. This study highlights an essential concept in eco-genomic biocontrol. Genomic potential is only one aspect of microbial function. A microorganism's ability to adapt flexibly to its environment may play a more important role in determining its effectiveness as a biocontrol agent.

Beyond biocontrol, Reddy et al. investigate how plant bio-stimulants affect productivity, bio-energy efficiency, and profitability in a berseem-pearl millet cropping system. The study examined treatments comprising plant growth-promoting rhizobacteria (PGPR), vesicular arbuscular mycorrhiza (VAM), seaweed extract, and different fertilizer levels under field conditions. Results showed that treatments combining PGPR and VAM with full recommended fertilizer, with or without seaweed extract, enhanced green and dry fodder yields, system-equivalent yield, energy output, energy productivity, and profitability. These findings highlight that microbial interventions can complement existing nutrient management practices rather than replace them. Importantly, the results suggest integrating microbial technologies into broader, more productive, and economically viable agricultural systems, where biological inputs provide measurable agronomic and financial benefits to support widespread adoption.

Bagul et al. highlight how host genotype and environment influence microbiome composition and disease resistance, using Ashwagandha as a case study in which microbiome profiling guides BCA discovery, illustrating that microbial solutions are context-dependent.

Finally, Pizzi et al. examine grapevine endophytes as sustainable biocontrol agents, showing how ecological compatibility and effective antagonism are crucial for practical application in vineyard management.

Overall, these studies suggest that future biocontrol will focus on modulating microbial functions and ecological interactions, integrating crop diversity, residue management, microbiome-based BCA discovery, genomic tools, and bio-stimulants within an eco-smart framework.

Challenges include environmental variability, soil and climate influences, crop-microbiome interactions, and long-term stability and safety concerns. Future priorities must include multi-location testing, long-term monitoring, standard efficacy criteria, formulation improvements, biosafety assessments, and economic analyses. Advanced omics and machine learning can further help identify microbial signatures associated with resilience and design tailored microbial consortia for specific contexts.

This Research Topic of studies advances a more ecological and systems-based perspective on biocontrol. By harnessing microbial communities, plant immune responses, soil biological processes, and the recycling of agricultural residues, eco-smart biocontrol can reduce reliance on synthetic inputs while enhancing productivity and resilience. Future sustainable agriculture will likely focus not on reducing microbial diversity, but on understanding, guiding, and responsibly utilizing these complexities.

